# Evaluation of physical and mental health status of orphan children living in orphanages in Sonitpur district of Assam: a cross-sectional study

**DOI:** 10.1186/s12887-022-03785-2

**Published:** 2022-12-19

**Authors:** Putul Mahanta, Kahua Das Thakuria, Pinky Goswami, Chandana Kalita, Ranjumoni Knower, Madhab Chandra Rajbangshi, Senjam Gojendra Singh, Jagadish Basumatary, Plabita Majumder

**Affiliations:** 1 Forensic Medicine and Toxicology, Assam Medical College, Dibrugarh, 786002 Assam India; 2 Physiology, Tezpur Medical College, Tezpur, 784010 Assam India; 3 Dentistry, Lakhimpur Medical College, 787001, Lakhimpur, Assam India; 4 Govt Dental College, 786002 Dibrugarh, Assam India; 5 Radiology, Fakhruddin Ali Ahmed Medical College and Hospital, Barpeta, Assam India; 6 Surgery, Tezpur Medical College, Tezpur, 784010 Assam India; 7 Psychiatry, Regional Institute of Medical Sciences, Imphal, 795004 India; 8 Anesthesiology, Tezpur Medical College, Tezpur, 784010 Assam India

**Keywords:** Balanced diet, BMI, Thinness, Homeless children, Mental health

## Abstract

**Background:**

Orphan children living in orphanages are often neglected. These children's physical and mental health status is essential as they are highly prone to malnourishment and psychosocial distress. We aim to evaluate the orphan children's physical and psychosocial status living in orphanages.

**Methods:**

This study adopted a cross-sectional research design conducted with the children living in the orphanages using a pretested, predesigned schedule. A total of 83 children (aged 5 to 19 years) living in three different orphanages in the Sonitpur District of Assam were randomly selected for the study. Body Mass Index (BMI) for age and height were then determined using WHO standards. Thinness was defined as BMI for age below -2 SD (Standard Deviation) and thinness as height for age below -2 SD. The behavioural and mental status of children aged 10–19 years were evaluated using the Strengths and Difficulties Questionnaire (SDQ-21) with a cut-off value of SDQ score > 15 as the presence of emotional and behavioural distress.

**Results:**

Almost 50% of orphans were in the age group of 10–14 years, 62.7% were females, and 42.2% had a primary level of education. 52.5% of orphans exhibited severe thinness for < -3 SD. Observed severe thinness more among the 5–9 years and 10–14 years (*p*-value < 0.05) group and among the male orphans (*p*-value < 0.05). Of 65 children aged 10–19, 18.5% had behavioural and mental distress. Emotional (32.3%) and poor conduct problems (23%) were observed significantly among male adolescents.

**Conclusions:**

Orphaned children, particularly those living in orphanages, are at risk of malnutrition and experience behavioural and psychosocial problems. Frequent assessments of their physical and mental health are advocated for early detection, prevention, and timely intervention.

## Background

An orphanage is a residential institution for taking care of and educating orphans. Children living in orphanages are a socially vulnerable group often neglected by mainstream society and are more prone to malnutrition [1].

About 24 million children worldwide live without their parents [2], out of which about 8 to 10 million are infants and children who live in orphanages [3]. In socioeconomically poor Asian countries, placing deprived children in orphanages has long been practised with few or no emotional and financial resources [4, 5].

Children in institutionalised care are continually in danger of undernutrition, overweight and micronutrient deficiencies [6], and they tend to be neglected [7]. Urban malnutrition is an increasing problem globally [8] and is more severe among children living in orphanages. Apart from malnutrition, children in orphanages also suffer from infectious diseases [9].

With its continuing concern for the health and well-being of children, particularly those existing in underprivileged living conditions, the United Nations Emergency Fund proposed a series of systematic studies under the auspices of different research agencies, including the social welfare ministry and the Government of India [4].

The most vulnerable children are those who have lost both parents. They lack the emotional and physical maturity to address their psychological trauma [5] and are at greater risk of developing depression and anxiety disorders [10]. Children living under institutional care often suffer from developmental and behavioural problems due to the lack of family care and support [11]. Various works suggest that the prevalence of mental and behavioural problems among those underprivileged children ranges from 18 to 60% [12–15]. The child's overall development is influenced by behavioural and mental problems, which may negatively impact their academic and social outcomes as adults [16].

Though limited in number, most of the orphanages in the Sonitpur district of Assam are in urban areas. Many of these orphans report various illnesses related to nutritional deficiency and are brought to the only tertiary care hospital in the Sonitpur district of Assam for different diseases. Also, research about those underprivileged children's behavioural and mental status is scarce in this region. Therefore, the present study evaluates the physical and psychosocial status of orphan children living in orphanages.

## Methods

A cross-sectional study was carried out at Tezpur Medical College and Hospital, Tezpur, Assam, among children aged 5 to 19 years living in orphanages in the Sonitpur district from October 2017 to October 2019. A pretested, predesigned questionnaire has been used for the data collection on the physical health of orphan children. A total of 83 orphan children living in three different orphanages of Sonitpur district meeting the inclusion criteria were randomly selected for the study.

The pretested and predesigned questionnaire included the participants' socio-demographic profile, anthropometric measurements, and physical and dental examination status. Anthropometric measurements were used to estimate the nutritional condition of teenagers. Height was recorded using a Stadiometer (Raja Industries, Agra, India) and weight by an electronic weight scale (Industrial weighing Machine (Activa Corporation, Tamil Nadu) wearing no shoes with light clothing. WHO guidelines were used to calculate BMI and height for age. Thinness was defined as BMI for age below -2 SD and stunting as height for age below -2 SD. Trained medical practitioners did physical health assessments for various nutritional disorders and dental health problems among the participants. Before implementation, the questionnaire was presented to an expert panel for validation. Also, it was pretested on four children each from the three orphanages, and further modifications were incorporated. To assess the behavioural and mental status of orphans of age ten years and above, the Strengths and Difficulties Questionnaire (SDQ self-report version) was used [17]. An SDQ score of > 15 was considered as the existence of emotional and behavioural problems.

The SDQ is a brief mental and behavioural problem screening questionnaire for children and adolescents. It comprises 25 attributes divided into five scales, i.e., emotional, conduct, hyperactivity/inattention, peer relationship, and pro-social behaviour, consisting of five items each. Several versions of the questionnaire are available to fulfil the needs of various users. There are SDQ versions for parents or educators of children aged 2–17 years and self-rated SDQ versions for 11–17-year-olds with or without impact supplements [18]. The impact supplements questions include chronicity, distress, social impairment, and burden to others, thus offering additional information for the observers. It has the advantage of a short format, is effective in identifying hyperactivity and inattention compared to other similar tools [17, 19], and is widely used globally [11, 20, 21]. Goodman et al. reported sensitivity of SDQ ranging from 81%-90% and specificity of 47% to 84% [22].

### Statistical analysis

The study findings were described through counts and percentages. To test the association between categorical variables, the Chi-square test was used. For small sample sizes with greater than 20% of cells having expected cell counts less than 5, Fisher exact test was performed for statistical significance testing, considering a *p*-value of less than 0.05 as significant.

### Ethics approval

Ethical clearance was obtained from the Institutional Human Ethical Committee of Tezpur Medical College and Hospital, Tezpur, Assam, India, with Ref: 20/17 dated 08/08/2017. Before collecting the data, informed consent was taken from the participant and the legal guardian in the case of a minor.

## Results

A large number of orphans (49.4%) were between the ages of 10–14, and 62.7% were represented by females with a primary level of education for 42.2% of participants, as shown in Table 1.

**Table 1:** Distribution of the orphans according to socio-demographic characteristics

Socio-demographic characteristics		No. of orphans (*n* = 83)	Percentage
Age (in years)	5—9	18	21.7
	10 -14	41	49.4
	15 -19	24	28.9
Gender	Male	31	37.3
	Female	52	62.7
Educational Level	Illiterate	11	13.2
	Primary School	35	42.2
	Middle School	13	15.7
	Drop-out	24	28.9

A relatively greater number, 32(38.6%) orphans, exhibited severe thinness (< -3SD). The height-for-age was normal (> -2SD) for all 83(100%) orphans, as shown in Table 2.

**Table 2:** Nutritional status of the orphans

Nutritional status of the orphans			No. of orphans (*n* = 83)	Percentage
BMI for Age	Severe thinness	< -3 SD	32	38.6
	Thinness	< -2 SD to -3 SD	24	28.9
	Normal	-2 SD to + 1 SD	27	32.5
	Overweight	> + 1 SD to + 2 SD	0	0
	Obesity	> + 2 SD	0	0
Height for age	Severely stunted	< -3 SD	0	0
	Stunted	< -2 SD to -3 SD	0	0
	Normal	> -2 SD	83	100

*BMI* Body Mass Index, *SD* Standard Deviation

Severe thinness was more among the orphans aged 5–9 years and 10–14 years than those in the 15–19 age group, which was found significant (*p*-value < 0.05). Severe thinness was also more among the male orphans than the females (*p*-value < 0.05), as shown in Table 3.

**Table 3:** Age and gender-wise distribution of thinness in orphans

Demographic Characteristics		Severe thinness^1^ (32)	Thinness^2^ (24)	Normal^3^ (27)	*p*-value
Age (In years)	5 -9 (*n* = 18)	11(61.1)	5(27.8)	2(11.1)	0.01
	10 -14 (*n* = 41)	18(43.9)	11(26.8)	12(29.3)	
	15 -19 (*n* = 24)	3(12.5)	8(33.3)	13(54.2)	
Gender	Male (*n* = 31)	21(80.0)	3(6.7)	7(13.3)	< 0.001
	Female (*n* = 52)	11(36.0)	21(20.0)	20(44.0)	

Figures in the brackets are the percentages; ^1^Severe thinness was defined as BMI for age below -3 SD; ^2^Thinness was defined as BMI for age < -2 SD to -3 SD; ^3^Normal defined as BMI for age -2 SD to + 1 SD

*BMI* Body Mass Index, *SD* Standard Deviation

On physical examination, pallor was detected in 4(5%) cases. During a clinical dental examination, 33(40%) orphans showed unhygienic dental habits like dental staining and dental caries in 4% of cases, besides other findings like calculus and decay, as shown in Table 4.

**Table 4:** Distribution of orphans as per the dental examination

Signs/Symptoms	Number of orphans (83)	Percentage
Normal	8	9.6
Enamel Stain	33	39.7
Malocclusion	27	32.5
Calculus	31	37.3
Gingival recession	11	13.2
Decay	16	19.3
Missing teeth	11	13.2
Irregular spacing	6	7.2
Dental caries	4	5.0
Upper anterior spacing	4	5.0

The behavioural and psychological conditions of the study children were assessed with SDQ. Out of 65 children of age ten years and above, 12(18.5%) had an SDQ score > 15, among whom the majority of 8(66.7%) children belonged to the 15–19 years age group (*p*-value = 0.018). Out of the 12 children having an SDQ score for positive psychosocial distress, 7(58.3%) were males. However, no statistically significant association was observed between the psychological condition of the children and gender, as shown in Table 5.

**Table 5:** Age and gender-wise distribution of SDQ score in orphans

Demographic Characteristics		SDQ Score > 15	SDQ Score ≤ 15	*p*-value
Age	10 -14 (*n* = 41)	4	37	0.018
	15 -19 (*n* = 24)	8	16	
Gender	Male (*n* = 24)	7	17	0.08
	Female (*n* = 41)	5	36	

**Fig. 1:**
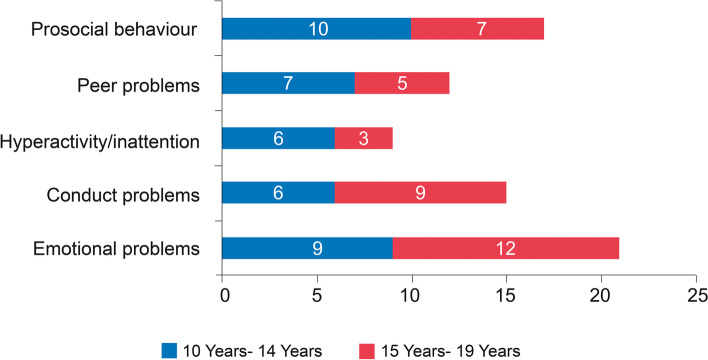
Distribution of orphans as per the SDQ subscales and age

Emotional problems were observed among 21(32.3%) children. Almost 26% of the children had poor pro-social behaviour, and 23% had conduct problems. Emotional (*p*-value = 0.019) and conduct problems (*p*-value = 0.03) were observed significantly more among the 15–19 years age group, while hyperactivity (p-value 0.8), poor pro-social behaviour (*p*-value = 0.18) and peer problems (*p*-value = 0.70) were mostly experienced among 10–14 years age group, as shown in Fig. 1.

**Fig. 2:**
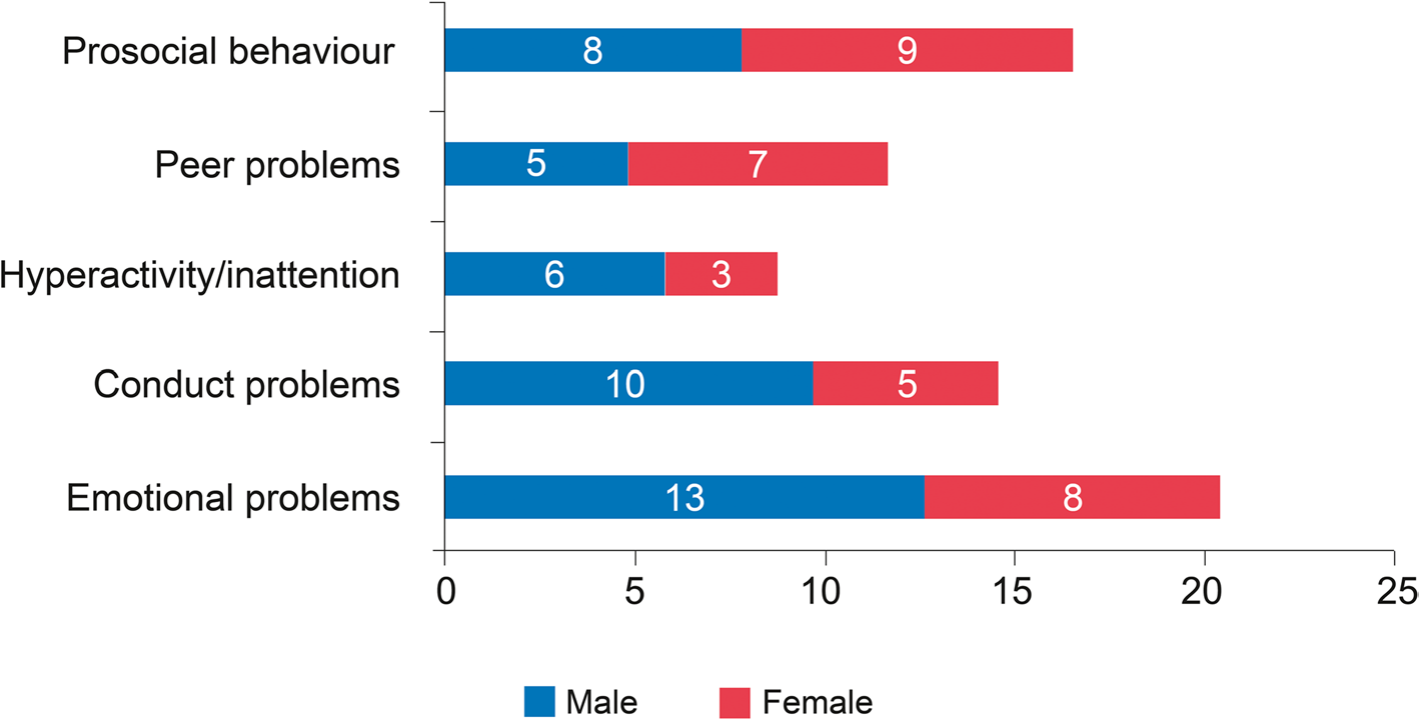
Distribution of orphans as per the SDQ subscales and age

Similarly, genderwise analysis showed that emotional problems (*p*-value = 0.004), conduct problems (*p*-value = 0.006) and hyperactivity or inattention (0.046) were significantly more among males, as shown in Fig. 2.

## Discussion

The result reports that a majority (49.4%) of orphans are in the 10–14 age group. Vaida N [4] also reported similar findings in his study.

The majority (38.6%) of the orphans were malnourished, reflected by severe thinness of < -3SD. Reddy SB et al. [1] also reported similar findings in their study on orphans. The same research has indicated that malnourishment is 57.7%, and stunting is 53.3% among orphan children. In their research, Chowdhury, ABMA et al. [7] mentioned about 60.03% of malnourishment among the studied children agree with the current findings**.**

Malnourishment, specifically undernutrition, was observed more among younger children than older children, supported a study [6]. There is a statistically significant association between thinness and age and gender; these findings were well tallied with some research investigations [1, 7].

The condition was normal concerning the height for age, as 100% of children were healthy in the current study. Similarly, as per Waterloo’s Classification in a survey of Vaida N [4], height for age was normal for more than half of the children studied.

On general physical examination, 79(95%) orphans were normal without any signs of pallor, found only in 4(5%) cases. The findings were similar to those reported in research [4]. The same research also supports the stained dental enamel observed in 40% of patients in the present study. In contrast to our findings, a study from Jammu and Kashmir reported nutrition-specific morbidities among 53% of the participants [23]. Another study from southern India reported poor physical health with multiple morbid conditions in 65% of the orphan children they studied [24, 25].

Dental caries in 5% of children in the present study were observed. It may be caused by the demineralisation of the enamel and the dentine by organic acids released from the sugars in the diet, supporting a study [26]. The organic acids increase the solubility of the calcium hydroxyapatite present in the teeth' hard tissue, resulting in demineralisation, as described in research [27].

Further, the results in the present study also revealed malocclusion in 32.5%, calculus in 37.3%, and gingival recession in 13.2%, besides decay in 19.3% of orphans, which were the signs of bad oral hygiene. The findings demonstrate a connection between diet and oral health, and the excellent state of oral health is correlated with a balanced diet. At the same time, incorrect nutritional intake relates to a form of oral disease. Some studies support these present findings [28–30]. A recent study conducted among children of various age groups living in orphanages in Kerala reported very high rates of dental caries, which contradicts our findings [31]. The same study suggested that those who had been shown by someone how to clean their teeth were 40% less likely to get caries [31].

In the present study, 18.5% of the 10–19 years old orphan children were contained with behavioural or mental distress. A study [11] reported similar findings in their research outcome. As per recent literature, the global prevalence of behavioural and emotional problems ranged from almost 18% to 65% among orphans and other susceptible children [11]. As these children are more exposed to abuse and mistreatment, often neglected in mainstream society and lack love and care, they are more prone to psychosocial distress.

Conduct and emotional issues were mainly found among orphans of adolescent age and males. A recent review [32] supports this result that orphan children are more prone to develop various behavioural and mental illnesses like depression, anxiety, and post-traumatic stress disorder. Like our research, multiple studies have cited age and gender as significant factors affecting the psychosocial status of orphan children [11, 32, 33]. Children growing up and living in orphanages have low social connections and risk a lack of education, and job opportunities are the reason for their various behavioural and mental illnesses. They are more prone to develop symptoms requiring psychopathological evaluations [34] to support the needs of this kind of research. A recent study observed that children out of parental care are more prone to develop behavioural disorders than those who live under parental care [35]. To lessen the burden of behavioural problems in orphan children living in orphanages, improvised institutional care is essential.

### Limitation

An average dietary recall of 24 h could have been considered though the shelter home did not permit it. It is a single-centre study. For better evaluation of different variables, a multicentre study comprising a large number of samples could have been initiated, which was not done in this study.

## Conclusion

Malnutrition is predominantly found in orphans living in orphanages, and this aspect of malnourishment needs to be analysed systematically and addressed scientifically. The children living in orphanages need to be checked frequently so that early detection, prevention, and timely intervention may improve their nutritional status.

A poor diet is significantly associated with increased oral diseases. Dietary advice for preventing oral diseases must be a part of routine patient education practices, mainly in shelter homes. Screening of these underprivileged children's mental and psychological status should be integrated with primary health care. Specifically, counselling and mental support should be advocated for healthy well-being among adolescents.

## Data Availability

The datasets used and/or analysed during the current study available from the corresponding author on reasonable request.

## Abbreviations

BMI: Body Mass Index
SD: Standard Deviation
SDQ: Strengths and Difficulties Questionnaire
